# Supplementary figures and images for: Lebanese cannabis oil extract protected against folic acid-induced kidney fibrosis in rats
Source: PLoS One. 2024 Dec 12;19(12):e0311790. doi: 10.1371/journal.pone.0311790 (PMC11637346; doi:10.1371/journal.pone.0311790)

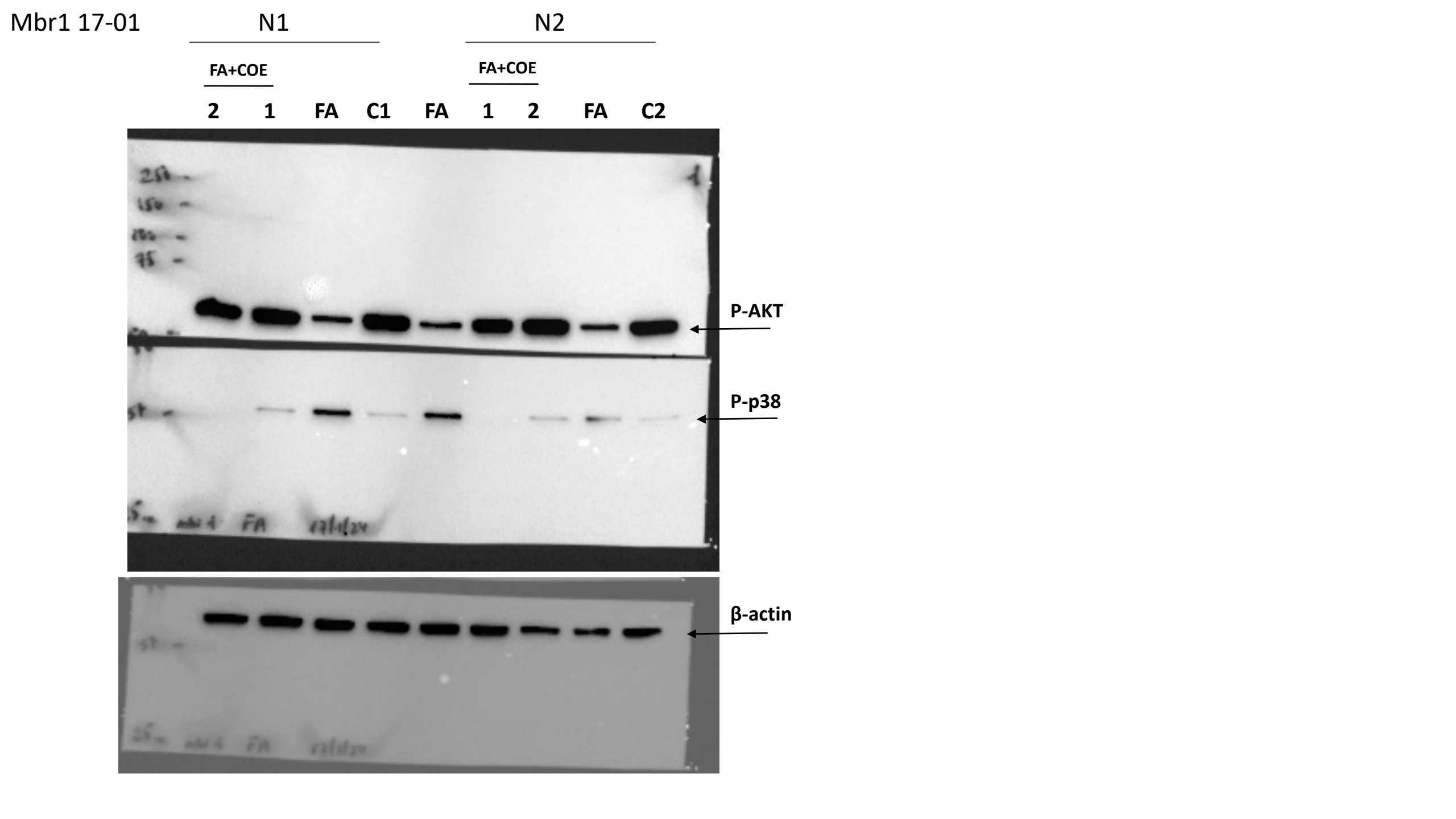

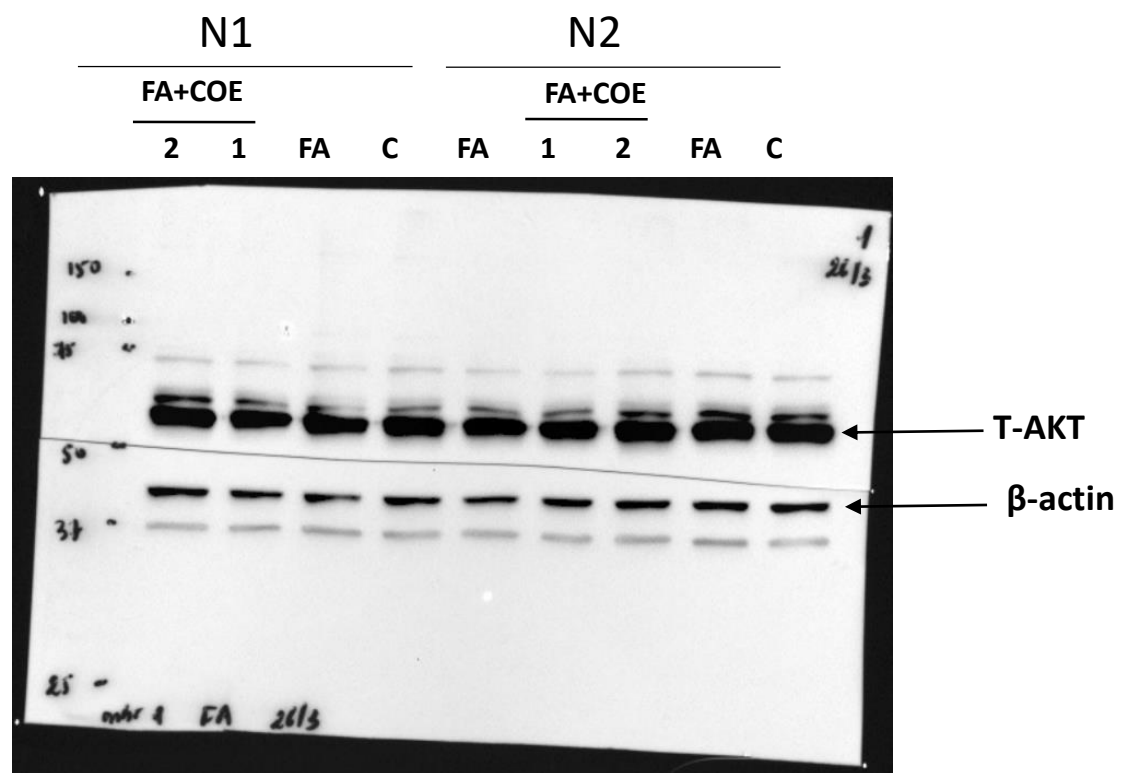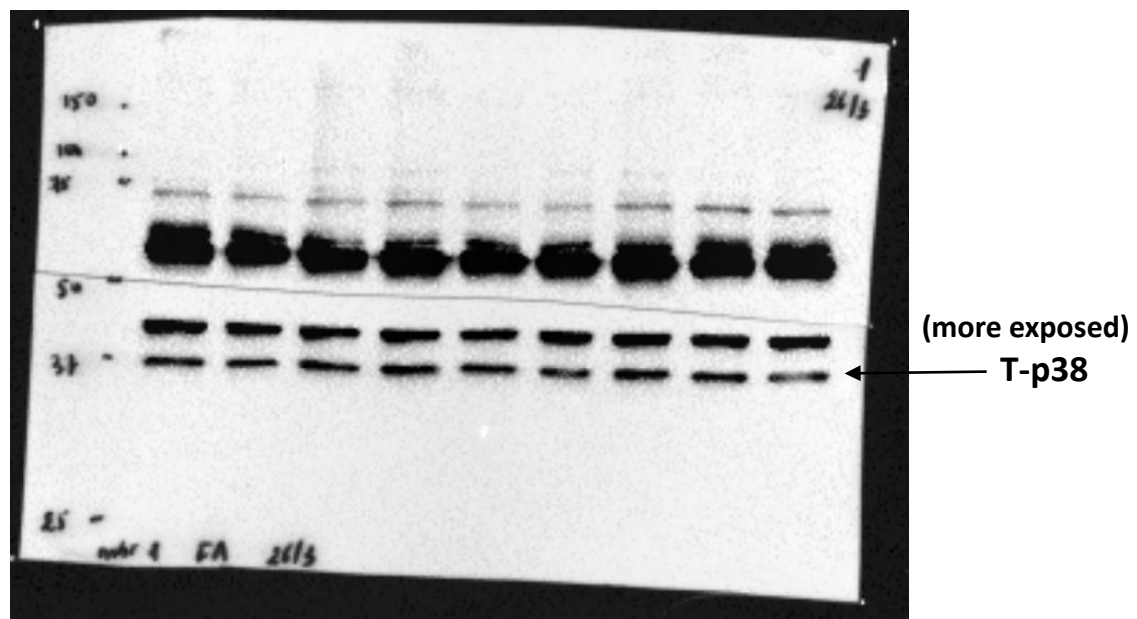

Mbr2 17-01

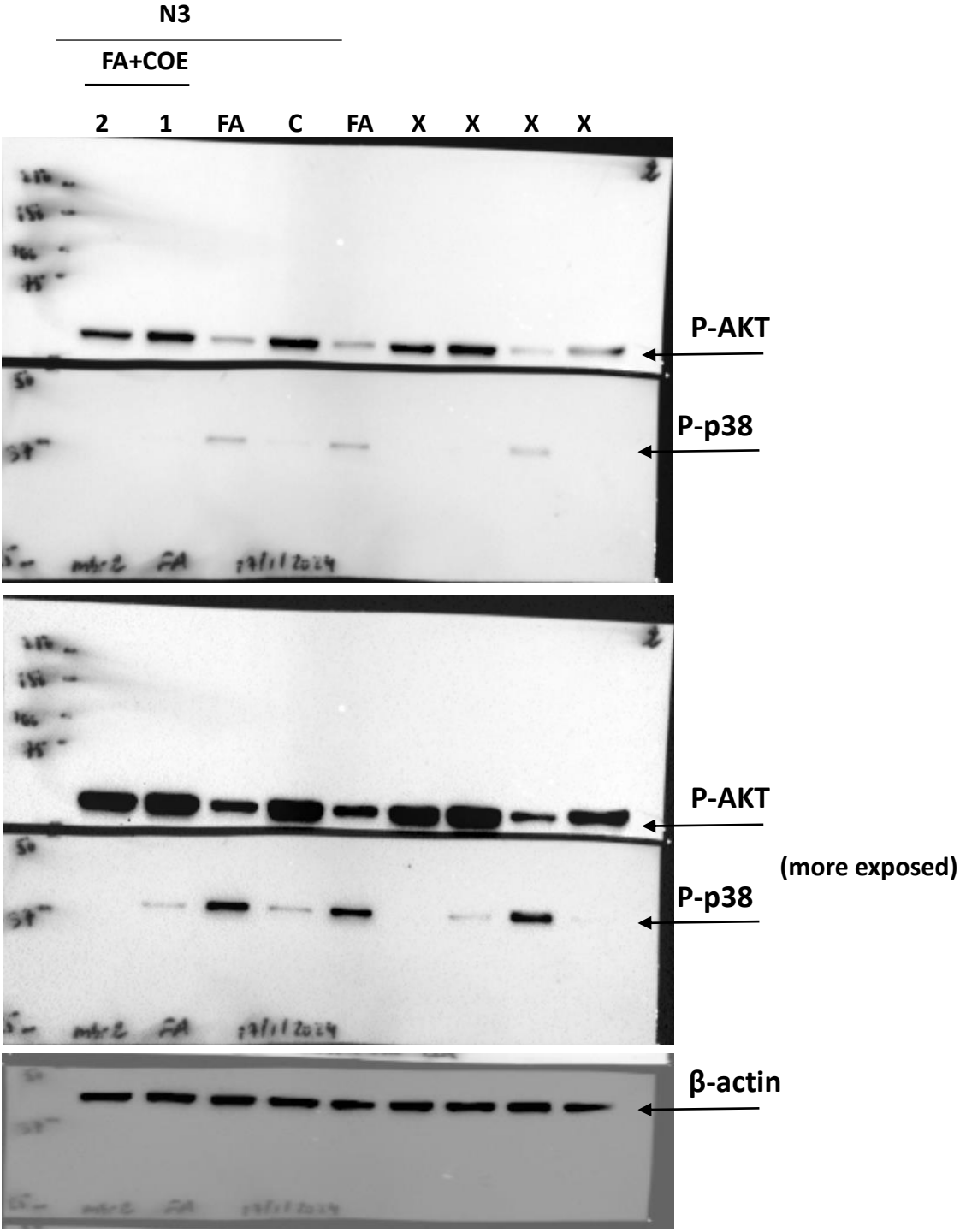

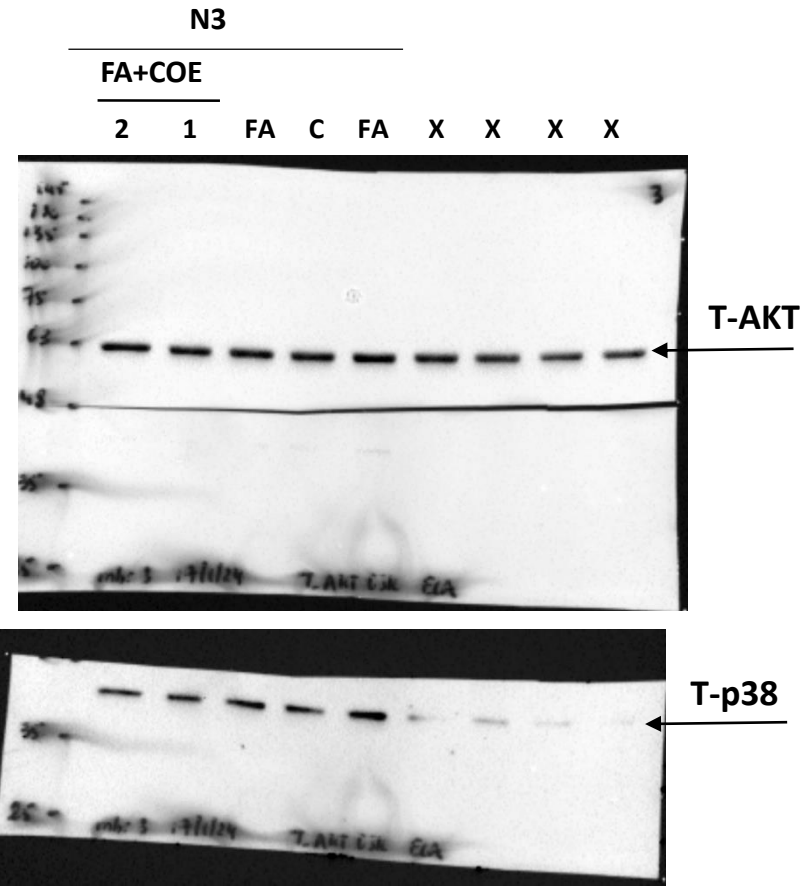

Supplement: S1 Raw images — (PDF) [file pone.0311790.s001.pdf]
