# Supplementary material for: Lebanese cannabis oil extract protected against folic acid-induced kidney fibrosis in rats
Source: PLoS One. 2024 Dec 12;19(12):e0311790. doi: 10.1371/journal.pone.0311790 (PMC11637346; doi:10.1371/journal.pone.0311790)
Supplement: Attachment — Submitted filename: Responses to reviewers.docx [file pone.0311790.s002.docx]

Dear Editor-in-Chief, PLoS One

We would like to thank you and the reviewers for their efforts in reviewing our manuscript and providing constructive comments. We answered each of the reviewers’ comments as described below and we made the necessary changes in the manuscript when applicable that can be found highlighted in red. Furthermore, we critically reviewed the paper for potential grammatical errors and made the necessary corrections

Reviewers' comments:

Reviewer's Responses to Questions

**Comments to the Author**

1. Is the manuscript technically sound, and do the data support the conclusions?

The manuscript must describe a technically sound piece of scientific research with data that supports the conclusions. Experiments must have been conducted rigorously, with appropriate controls, replication, and sample sizes. The conclusions must be drawn appropriately based on the data presented.

Reviewer #1: Yes

Reviewer #2: Yes

Reviewer #3: Yes

Answer: We thank the reviewers for their comments

2. Has the statistical analysis been performed appropriately and rigorously?

Reviewer #1: Yes

Reviewer #2: Yes

Reviewer #3: Yes

Answer: We thank the reviewers for their comments

3. Have the authors made all data underlying the findings in their manuscript fully available?

The [PLOS Data policy](http://www.plosone.org/static/policies.action#sharing) requires authors to make all data underlying the findings described in their manuscript fully available without restriction, with rare exception (please refer to the Data Availability Statement in the manuscript PDF file). The data should be provided as part of the manuscript or its supporting information, or deposited to a public repository. For example, in addition to summary statistics, the data points behind means, medians and variance measures should be available. If there are restrictions on publicly sharing data—e.g. participant privacy or use of data from a third party—those must be specified.

Reviewer #1: Yes

Reviewer #2: Yes

Reviewer #3: Yes

Answer: We thank the reviewers for their comments

4. Is the manuscript presented in an intelligible fashion and written in standard English?

PLOS ONE does not copyedit accepted manuscripts, so the language in submitted articles must be clear, correct, and unambiguous. Any typographical or grammatical errors should be corrected at revision, so please note any specific errors here.

Reviewer #1: Yes

Reviewer #2: Yes

Reviewer #3: Yes

Answer: We thank the reviewers for their comments

5. Review Comments to the Author

Please use the space provided to explain your answers to the questions above. You may also include additional comments for the author, including concerns about dual publication, research ethics, or publication ethics. (Please upload your review as an attachment if it exceeds 20,000 characters)

**Reviewer #1:** The manuscript deals with the effect of Lebanese Cannabis Oil Extract on Folic Acid-Induced Kidney Fibrosis.
It is well written and the methodology is rigorous. Some modifications are needed in order to improve it:
- Introduction: The sentence before the prevalence of dialysis in Lebanon has to be deleted. Only the prevalence has to be cited without announcing it.
- Discussion: The discussion section has to begin with the relevant results of the study.

**Answer to Reviewer 1**: we thank the reviewer for this comments. accorinlgy, we made the necessary changes as requested

**Reviewer #2:** The current work evaluated the protective effect of Lebanese cannabis oil extract against folic acid-induced renal injury both in vitro and in vivo. The authors have concluded that Lebanese cannabis oil extract significantly reduced serum creatinine in a rat model of folic acid-induced nephrotoxicity. The beneficial effect of Lebanese cannabis oil extract was also apparent in kidney and cardiac pathology examinations. Experimental design and statistical analysis are

Following are a few queries and suggestions.
1. Authors should support the results with good-quality photographs of cell culture showing wound healing.
2. The histological photomicrographs have been compared within the groups at different magnifications. Comparisons should be made at the same magnifications. The labelings on them are not clear.
3. Western blotting results should have been supported with immunofluorescence staining.

**Answer to Reviewer 2:**

We would to thank the reviewer for the comments:

1. We uploaded better quality photographs of cell culture of the wound healing
2. We changed the photos so that all photo show the same magnifications. Also, we adjusted the labeling to make it clearer. We also, made the necessary in the figures legends
3. We thank the reviewer for this comments. We used western blot to evaluate the phosphorylated activated levels of AKT and P38MAPK which is the most relevant in term of affecting cell survival. Although, immunostaining is important however, it will not provide a quantitative measurement of the activated kinase. It simply shows the localization of the kinase rather than its activated pattern. Furthermore, immunostaining of phosphorylated kinases is very challenging in particular when using paraffin embedded sections. Deparaffinization of paraffin section with xylene (a very strong organic solvent used to dissolve paraffin) prior staining with the relevant anti-phospho-protein antibody can cause a loss of the phospho group which bias the results. As such we preferred to do only western blot

**Reviewer #3:** Adverse effects of Cannabis, should be stated in the introduction section.

Figures 2, 7 & 8 are not clear (blurred, and dark). Please consider updating.

Discussion: Results were occasionally repeated without proper explanation or discussion.

**Answers to Reviewer 3**

We would to thank the reviewer for the comments. we uploaded better quality Figures 2, 7 & 8

We reviewed the discussion and made the necessary changes when applicable in addition to delete redundancies in the text

Respectfully,

Wissam, on behalf of all authors
